# Comparative safety profile of biktarvy: insights from a clinical cohort and the FAERS database

**DOI:** 10.3389/fphar.2026.1837474

**Published:** 2026-06-22

**Authors:** Xinmei Pan, Qingqing Tian, Qian Wang, Linli Xie, Jiangchuan Xie

**Affiliations:** 1 Department of Pharmacy, The First Affiliated Hospital of Army Medical University, Chongqing, China; 2 Department of Infectious Diseases, The First Affiliated Hospital of Army Medical University, Chongqing, China

**Keywords:** AES, biktarvy, disproportionality analysis, FAERS, HIV

## Abstract

**Background:**

Biktarvy, a second-generation integrase strand-transfer inhibitor, is approved for treating human immunodeficiency virus (HIV) in patients with no known resistance to its components. This study compared adverse events (AEs) associated with biktarvy based on data from the United States Food and Drug Administration Adverse Event Reporting System (FAERS) database and real-world evidence from a hospital setting to inform clinical practice.

**Methods:**

AE reports were extracted from the FAERS database and our hospital’s internal records. The Reporting Odds Ratio (ROR) and the Bayesian Confidence Propagation Neural Network (BCPNN) were used to assess the reporting of AEs associated with biktarvy. A positive signal was defined as meeting all of the following criteria: case number ≥ 3, ROR 95% confidence interval lower limit > 1.0, (IC-2SD) > 0.

**Results:**

A total of 10,424 AE reports were retrieved (6,850 from FAERS and 3,574 from hospital data). Males and patients aged 18–64.9 years accounted for a larger proportion of AE reports in terms of raw counts. Overall, 343 unique AEs met the positive signal threshold (263 from FAERS and 80 from the hospital). Hospital data identified signals including elevated urine vitamin C, triglycerides, and uric acid. FAERS data revealed signals for cerebrovascular accident, myocardial infarction, and cardiomegaly.

**Conclusion:**

Hospital data indicated a risk of eye disorders and certain abnormal investigation findings not mentioned in the package insert, while the FAERS data pointed to a potential risk domain of vascular diseases such as cerebrovascular accident and myocardial infarction. Our study provides evidence for the use of biktarvy in the treatment of HIV.

## Introduction

The treatment of human immunodeficiency virus (HIV) has significantly improved with the emergence of new antiretroviral drugs, especially second-generation integrase strand transfer inhibitors (INSTIs), which are characterized by higher genetic barriers to resistance, better efficacy and safety profiles, and fewer drug–drug interactions (Wei et al., 2023). Biktarvy (bictegravir sodium/emtricitabine/tenofovir alafenamide) is a low-dose, fixed-dose combination single-tablet regimen based on second-generation INSTIs. It not only incorporates the advantages of second-generation INSTIs, but also improves medication adherence among people living with HIV (PLWH), simplifies management, and enhances quality of life due to the characteristics of its compound formulation (Maggiolo et al., 2022). The clinical trial results have shown that the clinical efficacy of biktarvy is non-inferior to other fixed dose once daily combinations such as dolutegravir + emtricitabine (FTC) + tenofovir alafenamide (TAF) and dolutegravir + abacavir + lamivudine (De Clercq et al., 2023).

In February 2018, the U.S. Food and Drug Administration (FDA) was the first to approve biktarvy for the treatment of adults with human immunodeficiency virus type 1 infection, requiring patients to have no previous resistance to integrase inhibitor drugs, FTC, or TAF. Subsequently, biktarvy was approved in multiple countries and regions such as the European Union and China, and its applicable age range was continuously expanded. Especially in April 2025, the National Medical Products Administration of China approved the removal of drug restrictions on the use of FTC resistant population in Chinese instructions, supplemented clinical data on pregnant women and breastfeeding women, which further expanded the scope of application of biktarvy. In the United States, the clinical use of biktarvy has increased year by year from 2018 to 2020, with its overall utilization approaching that of the classic regimen dolutegravir/abacavir/lamivudine (Khazanchi et al., 2023). Guidelines list biktarvy as a first-line recommended regimen for HIV infection (Saag et al., 2020). Global clinical use of biktarvy is expected to continue increasing annually. In clinical trials, the most common adverse reactions of biktarvy (with an incidence rate greater than or equal to 5%) are diarrhea, nausea, and headache. However, with the expansion of the applicable population, the medication situation in the real world is far more complex than clinical trials. There are still many reports of new adverse events (AEs) of biktarvy, such as severe lactic acidosis and severe rhabdomyolysis (Simeni Njonnou et al., 2020; Parmar et al., 2023; Strike et al., 2024; Yokomaku et al., 2025). Although postmarketing AEs are voluntarily reported from an undefined population, the inherent limitations of spontaneous reporting systems — particularly the lack of denominator data and reporting biases — preclude accurate incidence estimation or definitive causal determination from these data alone. Nonetheless, such reports continue to support potential safety label updates. This study analyzed the AEs that occurred after the use of biktarvy based on two parts of data, a large post-marketing AE collection database [Food and Drug Administration Adverse Event Reporting System (FAERS)] and AEs recorded in the medical records of 283 Chinese PLWH (Potter et al., 2025). The database data and real-world clinical professional data (hospital data) complement and verify each other, increasing the credibility of the results. It is expected to further enrich the safety data of biktarvy and provide more reference information for complex clinical medication.

## Methods and materials

### Data source

Our study collected AE data for biktarvy from the FAERS Quarterly Data Extract Files and our hospital. FAERS data were collected from the first quarter of 2018 (FDA marketing approval of biktarvy for the treatment of HIV) to the first quarter of 2025 (the most recent update of the FAERS database at the time the study was performed). Hospital data were retrospectively extracted from the electronic medical record system of The First Affiliated Hospital of Army Medical University between the fourth quarter of 2021 (the earliest documented start date of biktarvy available in the hospital system) and the first quarter of 2025. AEs were identified from clinical notes, laboratory results, and discharge summaries. These data were not submitted to the national pharmacovigilance system. FAERS is a global spontaneous reporting system and does not include institutional patient identifiers. Our hospital data were derived from local electronic medical records. These two data sources are fully independent, with no patient overlap or double-counting. Our FAERS analysis is an exploratory hypothesis-generating signal detection study and does not constitute a definitive causal assessment.

### Drug identification

Reports involving biktarvy for HIV were identified in FAERS by both brand and generic names during the data mining process.

### Ethics approval

This study was approved by the Hospital Ethics Committee of the First Affiliated Hospital of Army Medical University ([B]KY2025280) and was performed in accordance with the Declaration of Helsinki.

### Exclusion criteria

Only events in our hospital meeting the following criteria were excluded: (a) events documented as pre-existing before biktarvy initiation; (b) events explicitly attributed by the treating physician to a cause other than biktarvy (e.g., trauma).

### Disproportionality analysis

As detailed in Supplementary Table S1, disproportionality analysis was performed using two complementary algorithms (the Reporting Odds Ratio [ROR] and the Bayesian Confidence Propagation Neural Network [BCPNN]) to enhance signal detection robustness. Calculations were performed using the contingency table calculator in OpenVigil 2.1. Both the ROR and BCPNN quantify disproportionate reporting rates against a background database to measure the strength of drug-AE associations, serving as screening tools for potential safety signals (Jiang et al., 2025). The FAERS data were deduplicated following standard FDA recommendations, retaining the most recent report for each unique Case ID (Potter et al., 2025). Our workflow is presented in Supplementary Figure S1. A positive signal was defined as meeting all of the following criteria: at least three cases, an ROR 95% confidence interval with a lower limit greater than 1.0, and an Information Component (IC) with its lower 95% confidence limit (IC-2SD) greater than 0 (the calculation formulas are shown in Supplementary Table S2). Time-to-onset was calculated after excluding cases with missing onset dates. All statistical analyses were conducted using MySQL (version 8.0) and R (version 9.1.0).

## Results

### Baseline characteristics of biktarvy

The baseline characteristics are summarized in Table 1, and the detailed data are shown in Supplementary Table S3. A total of 10,424 AE reports were retrieved: 6,850 from FAERS and 3,574 from our hospital (283 PLWH). Several notable demographic patterns were observed. As for gender, the absolute count of AE reports was higher in males than in females, with a male-to-female ratio of approximately 3:1 in both data sources. However, interpretation is limited by the absence of denominator data. In terms of age distribution, the majority of reports involved patients aged 18–64.9 years, constituting 48.0% of FAERS reports and 85.2% of hospital reports. A key difference lay in the reporters’ background: in FAERS, 30.2% of reports were submitted by consumers and 6.6% had missing reporter information, whereas 100.0% of the hospital reports came from healthcare professionals, which substantially enhanced the data completeness and reliability of the internal dataset. Furthermore, hospitalization was the most commonly reported serious outcome in FAERS (20.2%). Conversely, AEs documented in our hospital were generally less severe, with only 0.3% leading to hospitalization. Geographically, the FAERS reports predominantly originated from the United States. Temporally, the annual count of FAERS reports began to rise in 2019 and peaked in 2023. In contrast, reporting from our hospital started in 2021 and reached its peak in 2024.

**TABLE 1:** The key demographic characteristics of the study population.

Items	Number	Percentage	Number	Percentage
​	FAERS		Hospital	
Sex				
Female	1592	23.2%	69	24.4%
Male	4727	69.0%	214	75.6%
Missing	531	7.8%	0	0.0%
Weight				
<50 kg	106	1.5%	0	0.0%
50∼100 kg	594	8.7%	62	21.9%
>100 kg	78	1.1%	0	0.0%
Missing	6072	88.6%	221	78.1%
Age				
<18	171	2.5%	0	0.0%
18–64.9	3288	48.0%	241	85.2%
65–85	433	6.3%	42	14.8%
>85	12	0.2%	0	0.0%
Missing	2946	43.0%	0	0.0%

### Disproportionality analysis

**FIGURE 1:**
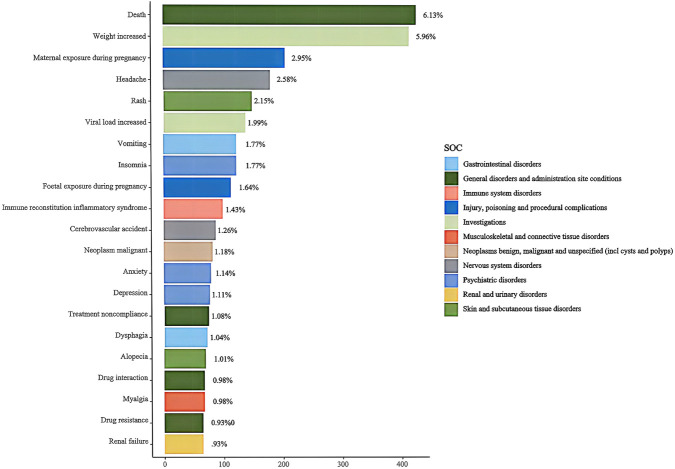
The top 20 Preferred Terms (PTs) reported in the FAERS database.

**FIGURE 2:**
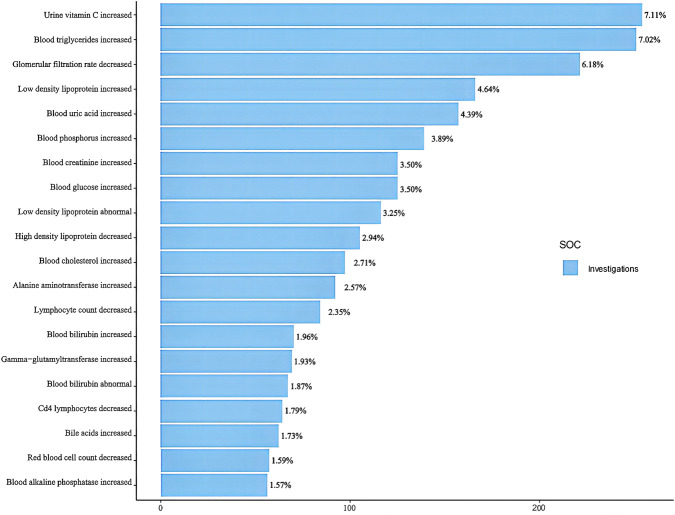
The top 20 Preferred Terms (PTs) observed in a hospital setting.

**FIGURE 3:**
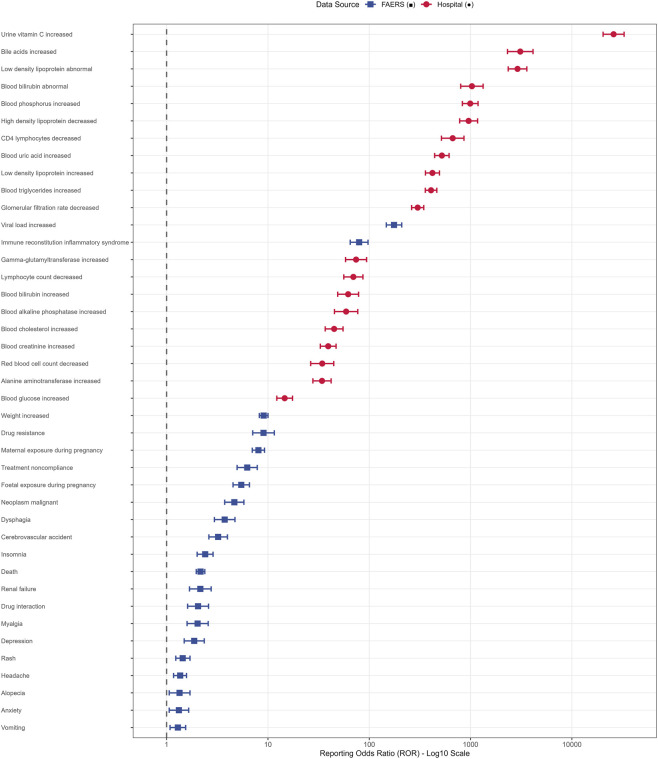
Forest plot comparing the reporting odds ratios (RORs) of the top 20 preferred terms (PTs) between the FAERS database and hospital data. Blue squares represent FAERS data, and red circles represent hospital data. Horizontal lines indicate 95% confidence intervals (CIs). The vertical dashed line at ROR = 1.0 indicates no disproportionate reporting; points to the right of this line suggest a positive signal. The x-axis is plotted on a logarithmic scale due to the wide range of ROR values (from approximately 1–30,000). PTs are ordered by the ROR magnitude from the hospital data.

As outlined in Supplementary Table S4, the included AEs were categorized by Preferred Terms (PTs) and System Organ Classes (SOCs). Figures 1, 2 showed the top 20 Preferred PTs and their corresponding SOC results in the FAERS database and hospital data, and Figure 3 presented the forest plot results for the top 20 PTs in both databases. Detailed data are shown in Supplementary Table S2. Subsequent disproportionality analysis identified AEs that were highly associated with biktarvy in both datasets. These signals collectively spanned nine SOCs: gastrointestinal disorders, hepatobiliary disorders, infections and infestations, injury, poisoning and procedural complications, investigations, metabolism and nutrition disorders, musculoskeletal and connective tissue disorders, neoplasms benign, malignant and unspecified (including cysts and polyps), and respiratory, thoracic and mediastinal disorders. A total of 343 unique PTs met the criteria for a positive signal, with 263 originating from FAERS and 80 from the hospital data. Importantly, 176 PTs from FAERS and 64 PTs from the hospital dataset were not documented in the prescribing information. Notably, the hospital dataset revealed signals for specific laboratory abnormalities, including increased urine vitamin C, elevated blood triglycerides, and high blood uric acid. In contrast, the FAERS database yielded signals for serious vascular events and cardiac events, namely cerebrovascular accident, myocardial infarction, and cardiomegaly.

### Time-to-onset analysis

**FIGURE 4:**
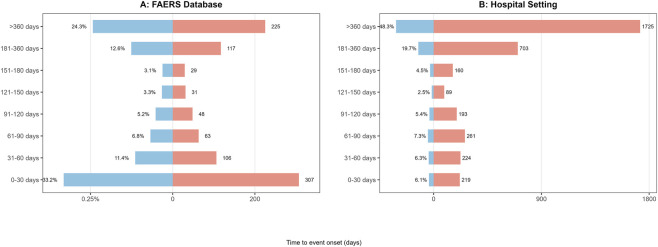
Time to onset of drug-related adverse events (AEs). **(A)** Time to onset of AEs in the FAERS database; **(B)** Time to onset of AEs in a hospital setting.

The time interval from biktarvy initiation to AEs onset was analyzed, and the results are presented in Table 2; Figure 4. Data completeness for the time-to-onset field was significantly higher in the hospital dataset (documented in 3,574 cases) than in FAERS (documented in 926 cases). The distribution of onset times differed markedly between the two sources. In the hospital data, AEs with later onset were predominant: 19.7% occurred between 181–360 days, and 48.3% occurred after 360 days. Conversely, in FAERS, early-onset AEs were most frequent: 33.2% occurred within 0–30 days, followed by 24.3% occurring after 360 days. Consistent with the distribution patterns, the median time to onset was 289.5 days (interquartile range [IQR], 148.75–430.25) for FAERS reports and 357 days (IQR, 125–631) for hospital cases.

**TABLE 2:** Onset of PTs induced by biktarvy in different data sources.

Group	Case number	Percentage	Case number	Percentage
Time-to-onset (days)	FAERS		Hospital	
0–30	307	33.2%	219	6.1%
31–60	106	11.4%	224	6.3%
61–90	63	6.8%	261	7.3%
91–120	48	5.2%	193	5.4%
121–150	31	3.3%	89	2.5%
151–180	29	3.1%	160	4.5%
181–360	117	12.6%	703	19.7%
>360	225	24.3%	1725	48.3%
Median time	289.5		357	
IQR	148.75–430.25		125–631	

## Discussion

To conduct an in-depth exploration of the real-world safety profile of biktarvy, this study performed a systematic analysis of its AE spectrum, and the demographic and clinical characteristics of PLWH experiencing AEs by integrating data from our hospital and the FAERS database. Our study is an exploratory hypothesis-generating signal detection study, not a definitive causal analysis. The FAERS and hospital data are independent sources with different reporting mechanisms. Thus, their event counts are not directly comparable. We found that the AEs highly correlated with biktarvy in both parts of the datasets coexisted in 9 SOCs through proportional imbalance analysis, and we identified 64 (hospital data) and 79 (FAERS data) unlabeled AE signals, respectively. Our hospital data suggested potential risks related to eye disorders and some abnormal laboratory findings, while the FAERS data pointed to potential risks in vascular disorders (e.g., cerebrovascular accident, myocardial infarction). These findings collectively contribute to the safety information of biktarvy.

### Feature analysis of AE

Analysis of the two datasets revealed the following commonalities and differences. First, the male-to-female ratio among PLWH experiencing AEs was highly similar (approximately 3:1), which is primarily associated with HIV transmission through male-to-male sexual behavior (Huang et al., 2015). Second, the two data sources differ fundamentally in reporting mechanisms: FAERS accepts spontaneous reports from any source (healthcare professionals: 63.2%), whereas all hospital AEs were directly identified by healthcare professionals through clinical assessments and laboratory monitoring, without reliance on patient self-reporting. While this high rate of professional reporting ensured the professionalism and reliability of the data, it also introduced a significant source of heterogeneity when comparing the two datasets. Healthcare professionals are more likely to report laboratory abnormalities, serious events, and clinically confirmed diagnoses, whereas consumer reports may over-represent subjective symptoms (e.g., headache, fatigue, insomnia) and under-represent asymptomatic laboratory findings or events requiring medical knowledge to recognize. This reporting bias may partially explain why the hospital dataset identified a larger number of abnormal investigation signals (e.g., urine vitamin C increased, blood triglycerides increased, blood uric acid increased), while FAERS contained more reports of subjective symptoms such as headache and nausea. Therefore, differences in AE signals between the two datasets should be interpreted with caution, as they may reflect reporting biases rather than true differences in the safety profile of biktarvy. Third, thanks to standardized medicine administration and follow-up, PLWH in our hospital generally had a good prognosis, with very few hospitalizations and no deaths. Finally, the temporal distribution trend aligned with the product lifecycle, with AEs in the FAERS database predominantly from the United States, with the number increasing since 2019 and peaking in 2023. In contrast, our hospital’s AEs began in 2021 and peaked in 2024 with the drug’s entry into China’s healthcare insurance, consistent with the “Weber effect” (Hoffman et al., 2014).

This study identified a signal for increased urine vitamin C in our hospital’s data. Vitamin C renal leak has been reported in the context of antiretroviral therapy (ART)-induced renal tubular dysfunction (Gara et al., 2012; Ebenuwa et al., 2023), and certain antiretroviral agents—particularly older tenofovir formulations (TDF)—are known to cause proximal tubular injury with subsequent urinary solute wasting, including vitamin C (Gara et al., 2012). Although TAF has a more favorable renal safety profile than TDF, the possibility of a similar, albeit milder, effect cannot be entirely excluded. If such renal wasting occurs, it would result in increased urine vitamin C and concurrent decrease in blood vitamin C. PLWH have an increased consumption and demand for vitamin C, due to increased oxidative stress, often resulting in a lower baseline level (Ebenuwa et al., 2023). Therefore, the renal leak that may be induced by drugs will overlap with this pathophysiological basis, further exacerbating the risk of vitamin C deficiency. Clinicians are advised to be alert to potential vitamin C deficiency in individuals receiving biktarvy treatment, and to promptly conduct nutritional assessments and dietary guidance.

Ocular complications are extremely common among PLWH, with an incidence rate as high as 70% (Chiotan et al., 2014). The main cause is opportunistic infections, but certain drug-induced nerve damage can also lead to ocular lesions (Riva et al., 2017). This study found that although the clinical trial data for biktarvy did not indicate the risk of ocular AEs, our hospital data observed AEs such as intraocular pressure test abnormal, anisometropia, and conjunctivitis. This discrepancy warrants further investigation. TAF and emtricitabine have been used clinically for many years without evidence of optic neurotoxicity. Our FAERS analysis generated a disproportionality signal for ocular AEs with biktarvy. This signal does not establish causality but warrants further investigation. Previous reports of ocular toxicity with elvitegravir—another integrase strand transfer inhibitor—raise the possibility of a class effect, but such extrapolation remains speculative (Riva et al., 2017). Confirmatory pharmacovigilance and mechanistic studies are needed. This may represent a novel mechanism worthy of further exploration. The epidemiological characteristics of HIV infection also vary with a country’s level of development (Lewallen and Courtright, 1997). The level of HIV diagnosis and treatment has rapidly improved, enabling the detection of previously overlooked ocular complications (Di et al., 2022). However, the technology to recognize and diagnose these complications is still imperfect, leading to misdiagnosis and missed diagnoses. This suggests that existing databases may not fully capture the real-world clinical picture within specific healthcare settings. Given that ocular lesions, especially retinal microvascular lesions, are important prognostic indicators, CD4^+^ T-lymphocyte counts are often used to predict the risk of ocular infections in PLWH (Thierfelder and Linnert, 1996). It is recommended that a decrease in CD4^+^ T-lymphocyte count be used as a warning signal to initiate routine ophthalmological screening, in order to achieve early intervention and protect PLWH’ vision (Martin-Odoom et al., 2016). This prospective monitoring is crucial for timely detection and management of drug-related or infectious ocular complications, and is essential for optimizing long-term HIV management and improving quality of life.

Among the PLWH in our hospital using biktarvy, the most frequently reported AEs were abnormalities in laboratory investigations. Some of these AEs are neither clearly documented in the product labeling nor reported in the FAERS database. Examples include triglycerides increased, uric acid increased, phosphorus increased, blood glucose increased, high density lipoprotein (HDL) decreased, lymphocyte count decreased, bile acids increased, red blood cell count decreased, and alkaline phosphatase (ALP) increased. Existing research has demonstrated that TAFand bictegravir (BIC) can contribute to weight gain (Hsu et al., 2024). The increase in triglycerides may be an indirect manifestation of TAF and BIC induced weight gain (Hsu et al., 2024), or it may also be a manifestation of dyslipidemia due to the “return-to-health effect” following HIV viral suppression (Mohr et al., 2018). A subgroup analysis of a clinical trial involving Japanese subjects receiving biktarvy revealed a statistically significant increase in triglycerides from baseline (Yokomaku et al., 2025). This suggests that the triglyceride levels of PLWH in Asia may require attention. Metabolic syndrome can significantly affect the uric acid levels in PLWH undergoing highly active ART (Pirro et al., 2018). The metabolic changes induced by biktarvy may also contribute to elevated uric acid. Currently, there are no published reports indicating that HIV treatment directly leads to elevated blood phosphorus, and the underlying mechanism remains unclear. However, the progression of kidney disease can result in elevated blood phosphorus. The high phosphorus phenomenon associated with biktarvy may still be related to the kidney damage caused by TAF, and the specific mechanism requires further investigation (Zirino et al., 2024). BIC may induce mitochondrial dysfunction, thereby disrupting normal carbohydrate metabolism and glucose utilization in hepatocytes and leading to elevated blood glucose (García-Martínez et al., 2024). This mechanism could further promote the development of insulin resistance and diabetes. Therefore, close monitoring of its impact on blood glucose is warranted in clinical application. The enhanced cellular immune function in PLWH is accompanied by an increase in HDL (Friis-Møller et al., 2003). A decrease in HDL may indicate a decline or disorder in the immune function of PLWH. Clinicians should comprehensively assess treatment efficacy and the overall metabolic-immune status of PLWH by integrating specific subgroup analysis indicators. The lymphocyte count can reflect the quantity and percentage of different types of white blood cells, and its reduction is one of the hallmark manifestations of HIV infection (Gao et al., 2023). In clinical treatment, lymphocyte count decreased may lead to more AEs and treatment interruption (Rockstroh et al., 2025). In PLWH, mucocutaneous lesions are correlated with lymphocyte count (Ashwini et al., 2020). Therefore, it is necessary to strengthen clinical monitoring of lymphocyte count. In recent years, there has been much attention paid to the mechanism of elevated bile acids. This mechanism posits that primary bile acids synthesized by hepatocytes enter the intestine, where they are metabolized by gut microbiota into secondary bile acids (Fuchs and Trauner, 2022). HIV infection itself or ART may affect the synthesis and absorption of secondary bile acids by altering gut microbiota and permeability, thereby interfering with hepatic bile acid circulation and synthesis feedback regulation through portal vein reflux, ultimately causing fluctuations in serum bile acid levels (Fuchs and Trauner, 2022; Enriquez et al., 2023). Red blood cells play an important role in the pathogenesis of HIV, and the decrease in their count is associated with factors such as depletion caused by HIV infection and nutritional deficiencies in PLWH (Motswaledi et al., 2013). Research analysis reveals that elevated ALP is most significantly associated with comorbidity status and demographic parameters of patients (Alemu et al., 2016). The unique hepatopathy background of the Chinese population may further amplify the risk of hepatotoxicity, leading to an increase in ALP (Zhou et al., 2020).

The AE reports collected in the FAERS database vary in quality due to differences in the backgrounds of the reporters. Among these, AEs with a large number of reports, such as maternal exposure during pregnancy, fetal exposure during pregnancy, and viral load increased, are clearly related to the infected individuals themselves or HIV infection. Nonetheless, the database contains numerous reports of common AEs documented in the package insert, such as weight increased, headache, rash, vomiting, and insomnia, which confirms that the database still has some utility. It is worth noting that we also observed AEs not mentioned in the package insert but deserving attention, namely, cerebrovascular accident and myocardial infarction. Both of them are manifestations of systemic atherosclerosis in different target organs, sharing common pathological mechanisms and risk factors (Arnett et al., 2019). Although the cardiovascular risk of PLWH is multifactorial, atherosclerosis is a major contributor to serious cardiovascular events such as myocardial infarction and cerebrovascular accident (Hansson et al., 2006). Our FAERS analysis identified a disproportionality signal for these events with biktarvy, which, while not establishing causality, raises the hypothesis that TAF may influence atherosclerosis progression (Schafer et al., 2019). This hypothesis requires confirmation through longitudinal studies and mechanistic research. Therefore, it is recommended that PLWH on long-term biktarvy therapy should pay attention to their systemic vascular health to facilitate early identification and prevention of related risks.

There is a discrepancy in the occurrence time of AE reported in the database and our hospital. The database reported AEs were mostly concentrated within 1 month and around 1 year after medication, and our hospital data showed that about half of the AEs occurred 1 year after medication. This difference may be related to the physical characteristics of different populations, such as the higher carrier rate of UGT1A1*28 allele in Caucasians, which may lead to delayed early metabolism of BIC and trigger more transient AEs (Geng et al., 2025). The completeness of AE induction time in the database accounts for only 13.5%. In contrast, PLWH in our hospital have regular follow-up and complete records, and the completeness of AE induction time accounts for as high as 100.0%. The data have more clinical reference significance. This also suggests that with prolonged medication time, there may be a risk of drug toxicity accumulation. Therefore, it is recommended that PLWH maintain regular follow-up and monitoring during long-term medication to timely identify and manage potential risks.

### Limitations

However, this study has limitations. First, FAERS data reflect reporting frequencies rather than prescription volumes, and our institutional data are single-center, which precludes formal pharmacoepidemiological inference about biktarvy utilization trends. Second, inherent biases of spontaneous reporting systems—including underreporting, selective reporting, and incomplete documentation—apply to FAERS (Gao et al., 2026). Furthermore, the absence of population exposure data prevents calculation of AE incidence or prevalence (Potter et al., 2025). Third, our hospital data were collected from a single center. This limits the generalizability of our findings to other populations with different genetic backgrounds, HIV subtype distributions, healthcare practices, and reporting cultures. Fourth, our study lacked a control group—such as HIV-positive patients receiving other antiretroviral regimens (e.g., dolutegravir-based therapies) or patients not exposed to biktarvy. Fifth, the two data sources differ substantially in reporter types (100% healthcare professionals in hospital data vs. 63.2% in FAERS), which limits the direct comparability of AE signals between them. Observed differences may reflect reporting behaviors rather than true differences in drug safety. Sixth, we did not systematically evaluate drug-drug interactions involving biktarvy and concomitant therapies, as FAERS lacks concomitant medication data, and our hospital analysis was exploratory in nature. Drug-drug interactions may influence AE occurrence and represent an important confounder not addressed in this study. Seventh, FAERS analyses were restricted to reports designating biktarvy as the Primary Suspect (PS). While this reduces confounding, it may miss true signals where biktarvy was coded as a secondary or concomitant suspect. Future studies should include all reporter-assigned roles for full signal coverage. These findings highlight the need for further research to better characterize the safety profile of biktarvy, particularly regarding the newly identified signals and their potential mechanisms.

## Conclusion

Our study utilized data from a hospital setting and the FAERS database to investigate AE signals associated with biktarvy in HIV treatment, thereby generating real-world evidence on its safety profile. While most identified AEs were consistent with the drug’s prescribing information, we also detected unlabeled signals: increased urine vitamin C, elevated blood triglycerides, and high blood uric acid in hospital data, and cerebrovascular accident, myocardial infarction, and cardiomegaly in FAERS data. Our triangulation design—combining FAERS signal detection with single-center electronic health record data—helps contextualize findings and partially mitigates limitations inherent to spontaneous reporting. To enable regulatory re-evaluation of the signals identified in this study, future pharmacovigilance research should prioritize: (1) independent validation using multi-center hospital data or regional registries; (2) active comparator studies comparing biktarvy with other integrase inhibitors (e.g., dolutegravir); and (3) linkage of FAERS signals with large-scale electronic health record databases to assess generalizability.

## Data Availability

The original contributions presented in the study are included in the article/Supplementary Material, further inquiries can be directed to the corresponding authors.
