# Correction: Comparative safety profile of biktarvy: insights from a clinical cohort and the FAERS database

**DOI:** 10.3389/fphar.2026.1934013

**Published:** 2026-07-22

**Authors:** Xinmei Pan, Qingqing Tian, Qian Wang, Linli Xie, Jiangchuan Xie

**Affiliations:** 1 Department of Pharmacy, The First Affiliated Hospital of Army Medical University, Chongging, China; 2 Department of Infectious Diseases, The First Affiliated Hospital of Army Medical University, Chongging, China

**Keywords:** AES, biktarvy, disproportionality analysis, FAERS, HIV

Authors Xinmei Pan, Qingqing Tian and Qian Wang were erroneously omitted as equal contributing author.

The original version of this article has been updated

