# Supplementary material for: Comparative safety profile of biktarvy: insights from a clinical cohort and the FAERS database
Source: Front Pharmacol. 2026 Jun 22;17:1837474. doi: 10.3389/fphar.2026.1837474 (PMC13333539; doi:10.3389/fphar.2026.1837474)
Supplement: Supplementary file 1 [file DataSheet1.docx]

**Supplementary Data**

**Table S1 Calculation of reporting odds ratio (ROR) and Bayesian confidence propagation neural network (BCPNN).**

| Items | Reports with target AE | Reports without target AE |
| --- | --- | --- |
| Reports with biktarvy | a | b |
| Reports without biktarvy | c | d |
| Total | a+c | b+d |

Abbreviations: a, number of reports containing the target drug (biktarvy) and target AE; b, number of reports containing other AEs of the biktarvy; c, number of reports containing the target AE of other drugs; d, number of reports containing other drugs and other AEs

**Table S2 Calculation formula and Principle of dis-proportionality measure and standard of signal detection.**

| Algorithms | Equation | Criteria |
| --- | --- | --- |
| ROR | ROR=ad/b/c | lower limit of 95% CI>1, N≥3 |
|  | 95%CI=e^ln(ROR)±1.96(1/a+1/b+1/c+1/d)^0.5^ |  |
| BCPNN | IC=log_2_a(a+b+c+d)(a+c)(a+b) | IC025>0 |
|  | 95%CI= E(IC) ± 2V(IC)^0.5 |  |

Abbreviations: ROR, The Reporting Odds Ratio; 95%CI, 95% confidence interval; N, the number of reports; BCPNN: the Bayesian Confidence Propagation Neural Network; IC, information component; IC025, the lower limit of 95% CI of the IC; E(IC), the IC expectations; V(IC), the variance of IC.

**Table S3 Characteristics of reports-all reports for biktarvy (2018 Q1 to 2025 Q1) .**

| Items | Number | Percentage | Number | Percentage |
| --- | --- | --- | --- | --- |
|  | FAERS | | Hospital | |
| Sex | | | | |
| Female | 1592 | 23.2% | 69 | 24.4% |
| Male | 4727 | 69.0% | 214 | 75.6% |
| Missing | 531 | 7.8% | 0 | 0.0% |
| Weight | | | | |
| ＜50 kg | 106 | 1.5% | 0 | 0.0% |
| 50～100 kg | 594 | 8.7% | 62 | 21.9% |
| ＞100 kg | 78 | 1.1% | 0 | 0.0% |
| Missing | 6072 | 88.6% | 221 | 78.1% |
| Age | | | | |
| <18 | 171 | 2.5% | 0 | 0.0% |
| 18-64.9 | 3288 | 48.0% | 241 | 85.2% |
| 65-85 | 433 | 6.3% | 42 | 14.8% |
| >85 | 12 | 0.2% | 0 | 0.0% |
| Missing | 2946 | 43.0% | 0 | 0.0% |
| Occupation of the reporter | | | | |
| Consumer | 2069 | 30.2% | 0 | 0.0% |
| Health Professional | 1321 | 19.3% | 1803 | 50.4% |
| Pharmacist | 1283 | 18.7% | 1771 | 49.6% |
| Physician | 1726 | 25.2% | 0 | 0.0% |
| Missing | 451 | 6.6% | 0 | 0.0% |
| Country of the reporter | | | | |
| US | 5080 | 74.2% | 0 | 0.0% |
| France | 274 | 4.0% | 0 | 0.0% |
| Portugal | 178 | 2.6% | 0 | 0.0% |
| Others | 1318 | 19.2% | 3574 | 100.0% |
| Serious cases or no Serious cases |  |  |  |  |
| NO | 2712 | 39.6% | 3563 | 99.7% |
| YES | 4138 | 60.4% | 11 | 0.3% |
| Fatal or no Fatal |  |  |  |  |
| NO | 6293 | 91.9% | 3574 | 100.0% |
| YES | 557 | 8.1% | 0 | 0.0% |
| Outcome |  |  |  |  |
| Congenital Anomaly | 93 | 1.4% | 0 | 0.0% |
| Death | 557 | 8.1% | 0 | 0.0% |
| Disability | 189 | 2.8% | 0 | 0.0% |
| Hospitalization | 1386 | 20.2% | 11 | 0.3% |
| Life-Threatening | 75 | 1.1% | 0 | 0.0% |
| Other | 4550 | 66.4% | 3563 | 99.7% |
| Year |  |  |  |  |
| 2018 | 306 | 4.5% | 0 | 0.0% |
| 2019 | 1023 | 14.9% | 0 | 0.0% |
| 2020 | 884 | 12.9% | 0 | 0.0% |
| 2021 | 983 | 14.4% | 1 | 0.0% |
| 2022 | 1126 | 16.4% | 511 | 14.3% |
| 2023 | 1187 | 17.3% | 1149 | 32.1% |
| 2024 | 1104 | 16.1% | 1467 | 41.0% |
| 2025 | 237 | 3.5% | 446 | 12.5% |

**Table S4 Signal strength of the preferred term (PT) and the clinical priority assessing results.**

| SOC | PT | N | ROR(95%Cl) | IC(IC025) | PT | N | ROR(95%Cl) | IC(IC025) |
| --- | --- | --- | --- | --- | --- | --- | --- | --- |
|  | FAERS | | | | Hospital | | | |
| blood and lymphatic system disorders | abdominal lymphadenopathy* | 4 | 15.97 ( 5.97 - 42.69 ) | 3.99 ( 2.69 ) | / | / | / | / |
| cardiac disorders | myocardial infarction* | 36 | 1.77 ( 1.28 - 2.46 ) | 0.82 ( 0.35 ) | / | / | / | / |
|  | cardiomegaly* | 5 | 2.92 ( 1.22 - 7.03 ) | 1.55 ( 0.37 ) | / | / | / | / |
| congenital, familial and genetic disorders | atrial septal defect* | 18 | 13.35 ( 8.4 - 21.23 ) | 3.73 ( 3.07 ) | / | / | / | / |
|  | ventricular septal defect* | 14 | 15.29 ( 9.04 - 25.86 ) | 3.92 ( 3.18 ) | / | / | / | / |
|  | trisomy 21* | 7 | 34.55 ( 16.38 - 72.9 ) | 5.09 ( 4.06 ) | / | / | / | / |
|  | patent ductus arteriosus* | 7 | 11.62 ( 5.53 - 24.42 ) | 3.53 ( 2.51 ) | / | / | / | / |
|  | polydactyly* | 5 | 22.09 ( 9.15 - 53.31 ) | 4.45 ( 3.27 ) | / | / | / | / |
|  | congenital hydronephrosis* | 4 | 27.92 ( 10.41 - 74.84 ) | 4.79 ( 3.49 ) | / | / | / | / |
|  | gene mutation* | 4 | 6.7 ( 2.51 - 17.87 ) | 2.74 ( 1.45 ) | / | / | / | / |
|  | haemophilia* | 3 | 61.49 ( 19.53 - 193.59 ) | 5.9 ( 4.44 ) | / | / | / | / |
|  | haemangioma congenital* | 3 | 54.6 ( 17.37 - 171.62 ) | 5.74 ( 4.27 ) | / | / | / | / |
|  | fallot's tetralogy* | 3 | 26.05 ( 8.35 - 81.3 ) | 4.69 ( 3.23 ) | / | / | / | / |
|  | drug resistance mutation* | 3 | 56.88 ( 18.09 - 178.87 ) | 5.79 ( 4.33 ) | / | / | / | / |
|  | bicuspid aortic valve* | 3 | 45.2 ( 14.41 - 141.74 ) | 5.47 ( 4.01 ) | / | / | / | / |
|  | deafness* | 18 | 3.06 ( 1.93 - 4.87 ) | 1.61 ( 0.95 ) | / | / | / | / |
| Eye disorders | / | / | / | / | intraocular pressure test abnormal* | 5 | 134.96(55.77-326.6) | 7.05(5.38) |
|  | / | / | / | / | anisometropia* | 4 | 2367.41(785.33-7136.6) | 10.87(9.08) |
|  | / | / | / | / | conjunctivitis* | 4 | 3.25(1.22-8.65) | 1.7(0.03) |
| endocrine disorders | graves' disease | 4 | 5.73 ( 2.15 - 15.28 ) | 2.51 ( 1.22 ) | / | / | / | / |
| gastrointestinal disorders | vomiting | 121 | 1.29 ( 1.08 - 1.54 ) | 0.36 ( 0.1 ) | gastritis* | 6 | 4.79(2.15-10.66) | 2.26(0.59) |
|  | dysphagia* | 71 | 3.74 ( 2.96 - 4.72 ) | 1.9 ( 1.55 ) | anal abscess* | 4 | 11.11(4.17-29.65) | 3.47(1.8) |
|  | abdominal distension | 46 | 2.19 ( 1.64 - 2.93 ) | 1.13 ( 0.71 ) | intestinal polyp* | 3 | 37.45(12.04-116.44) | 5.22(3.55) |
|  | pancreatitis* | 39 | 4.57 ( 3.33 - 6.25 ) | 2.19 ( 1.73 ) | / | / | / | / |
|  | flatulence | 23 | 2.06 ( 1.37 - 3.1 ) | 1.04 ( 0.45 ) | / | / | / | / |
|  | pancreatitis acute* | 21 | 4.83 ( 3.15 - 7.42 ) | 2.27 ( 1.65 ) | / | / | / | / |
|  | gastrointestinal pain* | 6 | 2.42 ( 1.08 - 5.38 ) | 1.27 ( 0.18 ) | / | / | / | / |
|  | inflammatory bowel disease* | 5 | 4.15 ( 1.72 - 9.97 ) | 2.05 ( 0.87 ) | / | / | / | / |
|  | oesophageal stenosis* | 4 | 7.17 ( 2.69 - 19.14 ) | 2.84 ( 1.54 ) | / | / | / | / |
|  | faeces pale | 3 | 7.2 ( 2.32 - 22.37 ) | 2.84 ( 1.4 ) | / | / | / | / |
|  | acute abdomen* | 3 | 11.89 ( 3.82 - 36.98 ) | 3.56 ( 2.12 ) | / | / | / | / |
|  | pancreatitis necrotising* | 3 | 6.05 ( 1.95 - 18.79 ) | 2.59 ( 1.15 ) | / | / | / | / |
|  | pancreatitis chronic* | 3 | 7.22 ( 2.32 - 22.44 ) | 2.85 ( 1.4 ) | / | / | / | / |
| general disorders and administration site conditions | death | 420 | 2.16 ( 1.96 - 2.38 ) | 1.09 ( 0.94 ) | / | / | / | / |
|  | treatment noncompliance* | 74 | 6.24 ( 4.96 - 7.84 ) | 2.63 ( 2.3 ) | / | / | / | / |
|  | drug interaction | 67 | 2.04 ( 1.61 - 2.59 ) | 1.02 ( 0.67 ) | / | / | / | / |
|  | drug resistance | 64 | 9.04 ( 7.07 - 11.56 ) | 3.17 ( 2.81 ) | / | / | / | / |
|  | unevaluable event* | 33 | 2.22 ( 1.58 - 3.13 ) | 1.15 ( 0.65 ) | / | / | / | / |
|  | fat tissue increased* | 11 | 33.7 ( 18.58 - 61.13 ) | 5.05 ( 4.21 ) | / | / | / | / |
|  | terminal state* | 9 | 6.63 ( 3.45 - 12.76 ) | 2.72 ( 1.81 ) | / | / | / | / |
|  | hunger* | 6 | 2.45 ( 1.1 - 5.45 ) | 1.29 ( 0.2 ) | / | / | / | / |
|  | disease complication* | 5 | 5.28 ( 2.2 - 12.71 ) | 2.4 ( 1.22 ) | / | / | / | / |
|  | chronic fatigue syndrome* | 3 | 8.93 ( 2.87 - 27.76 ) | 3.15 ( 1.71 ) | / | / | / | / |
| hepatobiliary disorders | liver injury* | 20 | 3.29 ( 2.12 - 5.11 ) | 1.72 ( 1.09 ) | hepatic function abnormal | 11 | 5.36(2.97-9.69) | 2.42(0.75) |
|  | liver disorder | 20 | 2.18 ( 1.4 - 3.37 ) | 1.12 ( 0.49 ) | cholestasis* | 6 | 5.34(2.4-11.9) | 2.41(0.75) |
|  | hepatic steatosis | 16 | 4.32 ( 2.64 - 7.06 ) | 2.11 ( 1.41 ) | hepatic fibrosis* | 3 | 16.92(5.45-52.53) | 4.08(2.41) |
|  | hepatitis | 15 | 2.84 ( 1.71 - 4.71 ) | 1.5 ( 0.78 ) | fatty liver* | 3 | 37.66(12.11-117.1) | 5.23(3.56) |
|  | hepatic failure | 14 | 2.7 ( 1.6 - 4.57 ) | 1.43 ( 0.69 ) | / | / | / | / |
|  | jaundice* | 13 | 3.11 ( 1.8 - 5.36 ) | 1.63 ( 0.86 ) | / | / | / | / |
|  | hepatic cirrhosis | 12 | 3.07 ( 1.74 - 5.41 ) | 1.62 ( 0.81 ) | / | / | / | / |
|  | hepatic cytolysis* | 12 | 3.06 ( 1.74 - 5.4 ) | 1.61 ( 0.81 ) | / | / | / | / |
|  | hyperbilirubinaemia* | 9 | 4.19 ( 2.18 - 8.05 ) | 2.06 ( 1.15 ) | / | / | / | / |
|  | hepatic pain* | 4 | 4.51 ( 1.69 - 12.04 ) | 2.17 ( 0.88 ) | / | / | / | / |
|  | ocular icterus | 3 | 4.35 ( 1.4 - 13.5 ) | 2.12 ( 0.67 ) | / | / | / | / |
|  | hepatic cyst* | 3 | 4.6 ( 1.48 - 14.27 ) | 2.2 ( 0.75 ) | / | / | / | / |
|  | portal hypertension* | 3 | 5.07 ( 1.63 - 15.74 ) | 2.34 ( 0.89 ) | / | / | / | / |
|  | mixed liver injury* | 3 | 3.67 ( 1.18 - 11.38 ) | 1.87 ( 0.43 ) | / | / | / | / |
|  | hepatitis toxic | 3 | 6.4 ( 2.06 - 19.89 ) | 2.67 ( 1.23 ) | / | / | / | / |
| immune system disorders | immune reconstitution inflammatory syndrome | 98 | 79.43 ( 64.9 - 97.22 ) | 6.25 ( 5.96 ) | / | / | / | / |
| infections and infestations | hiv infection | 40 | 67.94 ( 49.58 - 93.09 ) | 6.04 ( 5.58 ) | respiratory tract infection* | 15 | 9.51(5.72-15.79) | 3.24(1.58) |
|  | viraemia* | 38 | 94 ( 67.93 - 130.08 ) | 6.49 ( 6.02 ) | rhinitis* | 10 | 21.32(11.45-39.69) | 4.41(2.74) |
|  | tuberculosis | 28 | 10.06 ( 6.94 - 14.59 ) | 3.32 ( 2.78 ) | syphilis* | 10 | 194.16(103.68-363.61) | 7.57(5.89) |
|  | pneumocystis jirovecii pneumonia | 25 | 8.76 ( 5.91 - 12.98 ) | 3.12 ( 2.56 ) | pharyngitis* | 7 | 10.29(4.9-21.61) | 3.36(1.69) |
|  | virologic failure | 22 | 15.77 ( 10.37 - 24 ) | 3.97 ( 3.36 ) | otitis media chronic* | 5 | 233.69(96.1-568.27) | 7.83(6.15) |
|  | hepatitis c | 20 | 9.81 ( 6.32 - 15.23 ) | 3.29 ( 2.66 ) | prostatitis* | 3 | 20.2(6.5-62.75) | 4.33(2.66) |
|  | syphilis* | 19 | 94.49 ( 59.7 - 149.55 ) | 6.5 ( 5.84 ) | oral fungal infection* | 3 | 22.15(7.13-68.81) | 4.46(2.8) |
|  | monkeypox* | 17 | 192.61 ( 117.37 - 316.08 ) | 7.47 ( 6.76 ) | / | / | / | / |
|  | hiv viraemia* | 13 | 175.13 ( 99.6 - 307.94 ) | 7.34 ( 6.54 ) | / | / | / | / |
|  | hepatitis b | 11 | 12.01 ( 6.64 - 21.72 ) | 3.58 ( 2.74 ) | / | / | / | / |
|  | progressive multifocal leukoencephalopathy* | 11 | 6.25 ( 3.46 - 11.29 ) | 2.64 ( 1.8 ) | / | / | / | / |
|  | acquired immunodeficiency syndrome | 10 | 153.8 ( 81.05 - 291.85 ) | 7.17 ( 6.27 ) | / | / | / | / |
|  | meningitis cryptococcal* | 10 | 25.52 ( 13.68 - 47.6 ) | 4.66 ( 3.78 ) | / | / | / | / |
|  | cerebral toxoplasmosis* | 9 | 42.76 ( 22.11 - 82.71 ) | 5.39 ( 4.47 ) | / | / | / | / |
|  | mycobacterium avium complex infection | 8 | 12.56 ( 6.27 - 25.18 ) | 3.64 ( 2.68 ) | / | / | / | / |
|  | helicobacter infection* | 8 | 2.66 ( 1.33 - 5.31 ) | 1.41 ( 0.44 ) | / | / | / | / |
|  | pulmonary tuberculosis | 8 | 10.77 ( 5.37 - 21.57 ) | 3.42 ( 2.46 ) | / | / | / | / |
|  | coronavirus infection * | 7 | 2.42 ( 1.15 - 5.08 ) | 1.27 ( 0.25 ) | / | / | / | / |
|  | opportunistic infection | 7 | 19.01 ( 9.03 - 40 ) | 4.24 ( 3.21 ) | / | / | / | / |
|  | hiv-associated neurocognitive disorder* | 7 | 185.23 ( 85.72 - 400.28 ) | 7.42 ( 6.36 ) | / | / | / | / |
|  | hepatitis b reactivation | 6 | 5.42 ( 2.43 - 12.07 ) | 2.43 ( 1.34 ) | / | / | / | / |
|  | encephalitis* | 6 | 3.86 ( 1.73 - 8.59 ) | 1.94 ( 0.85 ) | / | / | / | / |
|  | chlamydial infection* | 6 | 44.76 ( 19.95 - 100.44 ) | 5.46 ( 4.35 ) | / | / | / | / |
|  | subcutaneous abscess* | 5 | 5.08 ( 2.11 - 12.22 ) | 2.34 ( 1.16 ) | / | / | / | / |
|  | disseminated tuberculosis | 5 | 10.71 ( 4.45 - 25.79 ) | 3.41 ( 2.23 ) | / | / | / | / |
|  | atypical mycobacterial infection* | 5 | 15.67 ( 6.5 - 37.77 ) | 3.96 ( 2.78 ) | / | / | / | / |
|  | meningitis* | 5 | 3.8 ( 1.58 - 9.13 ) | 1.92 ( 0.74 ) | / | / | / | / |
|  | histoplasmosis disseminated* | 4 | 10.57 ( 3.96 - 28.23 ) | 3.4 ( 2.1 ) | / | / | / | / |
|  | pneumocystis jirovecii infection | 4 | 12.36 ( 4.63 - 33.04 ) | 3.62 ( 2.33 ) | / | / | / | / |
|  | endocarditis* | 4 | 3.65 ( 1.37 - 9.74 ) | 1.87 ( 0.57 ) | / | / | / | / |
|  | gonorrhoea* | 4 | 60.67 ( 22.47 - 163.79 ) | 5.89 ( 4.57 ) | / | / | / | / |
|  | necrotising fasciitis* | 4 | 5.07 ( 1.9 - 13.52 ) | 2.34 ( 1.05 ) | / | / | / | / |
|  | toxoplasmosis* | 3 | 11.85 ( 3.81 - 36.85 ) | 3.56 ( 2.11 ) | / | / | / | / |
|  | mycobacterial infection | 3 | 7.09 ( 2.28 - 22.02 ) | 2.82 ( 1.38 ) | / | / | / | / |
|  | sexually transmitted disease* | 3 | 26.87 ( 8.61 - 83.88 ) | 4.73 ( 3.28 ) | / | / | / | / |
|  | disseminated mycobacterium avium complex infection | 3 | 40.15 ( 12.82 - 125.74 ) | 5.3 ( 3.84 ) | / | / | / | / |
|  | cryptosporidiosis infection* | 3 | 20.68 ( 6.64 - 64.47 ) | 4.36 ( 2.91 ) | / | / | / | / |
| injury, poisoning and procedural complications | maternal exposure during pregnancy | 202 | 8.05 ( 7 - 9.25 ) | 2.99 ( 2.78 ) | lumbar vertebral fracture* | 5 | 19.34(8.04-46.53) | 4.27(2.6) |
|  | foetal exposure during pregnancy | 112 | 5.45 ( 4.52 - 6.56 ) | 2.43 ( 2.16 ) | / | / | / | / |
|  | accident at work* | 4 | 9.04 ( 3.38 - 24.13 ) | 3.17 ( 1.88 ) | / | / | / | / |
|  | jaw fracture* | 3 | 7.85 ( 2.53 - 24.4 ) | 2.97 ( 1.52 ) | / | / | / | / |
| investigations | weight increased | 408 | 9.07 ( 8.22 - 10.01 ) | 3.14 ( 2.99 ) | urine vitamin c increased* | 254 | 25795.22(20310.32-32761.34) | 12.66(10.99) |
|  | viral load increased* | 136 | 175.59 ( 147.37 - 209.2 ) | 7.34 ( 7.08 ) | blood triglycerides increased* | 251 | 407.41(357.45-464.35) | 8.51(6.84) |
|  | cd4 lymphocytes decreased* | 61 | 160.73 ( 123.91 - 208.49 ) | 7.22 ( 6.85 ) | glomerular filtration rate decreased | 221 | 300.76(261.96-345.32) | 8.1(6.43) |
|  | blood hiv rna increased* | 54 | 105.2 ( 80.05 - 138.27 ) | 6.65 ( 6.25 ) | low density lipoprotein increased | 166 | 420.6(358.73-493.14) | 8.58(6.92) |
|  | blood creatinine increased | 50 | 3.91 ( 2.96 - 5.16 ) | 1.96 ( 1.56 ) | blood uric acid increased* | 157 | 521.15(442.22-614.16) | 8.88(7.21) |
|  | genotype drug resistance test positive | 38 | 138.01 ( 99.43 - 191.55 ) | 7.02 ( 6.54 ) | blood phosphorus increased* | 139 | 992.61(830.66-1186.15) | 9.75(8.08) |
|  | hepatic enzyme increased | 26 | 1.66 ( 1.13 - 2.44 ) | 0.73 ( 0.18 ) | blood creatinine increased | 125 | 39.32(32.88-47.02) | 5.24(3.57) |
|  | viral load abnormal* | 26 | 538.63 ( 351.18 - 826.13 ) | 8.76 ( 8.16 ) | blood glucose increased* | 125 | 14.62(12.23-17.48) | 3.82(2.15) |
|  | liver function test increased | 25 | 3.68 ( 2.49 - 5.45 ) | 1.88 ( 1.31 ) | low density lipoprotein abnormal | 116 | 2904.82(2351.12-3588.93) | 11.06(9.39) |
|  | blood creatine phosphokinase increased | 25 | 5.55 ( 3.74 - 8.22 ) | 2.47 ( 1.9 ) | high density lipoprotein decreased* | 105 | 957.36(780.86-1173.76) | 9.72(8.05) |
|  | glomerular filtration rate decreased | 25 | 8.2 ( 5.54 - 12.15 ) | 3.03 ( 2.46 ) | blood cholesterol increased | 97 | 45.02(36.77-55.11) | 5.45(3.78) |
|  | transaminases increased | 23 | 4.71 ( 3.12 - 7.09 ) | 2.23 ( 1.64 ) | alanine aminotransferase increased | 92 | 34.16(27.76-42.04) | 5.05(3.39) |
|  | blood cholesterol increased | 18 | 2.09 ( 1.31 - 3.31 ) | 1.06 ( 0.4 ) | lymphocyte count decreased* | 84 | 69.69(56.08-86.6) | 6.08(4.41) |
|  | blood triglycerides increased* | 17 | 6.59 ( 4.09 - 10.61 ) | 2.71 ( 2.03 ) | blood bilirubin increased | 70 | 61.89(48.81-78.47) | 5.91(4.25) |
|  | blood bilirubin increased | 15 | 3.34 ( 2.01 - 5.54 ) | 1.74 ( 1.01 ) | gamma-glutamyltransferase increased | 69 | 74.12(58.35-94.16) | 6.17(4.51) |
|  | glycosylated haemoglobin increased* | 13 | 2.23 ( 1.29 - 3.84 ) | 1.15 ( 0.38 ) | blood bilirubin abnormal | 67 | 1031.49(799.39-1330.98) | 9.83(8.16) |
|  | hiv test positive | 10 | 96.04 ( 51 - 180.86 ) | 6.53 ( 5.64 ) | cd4 lymphocytes decreased* | 64 | 666.52(515.97-861.01) | 9.25(7.58) |
|  | blood lactic acid increased | 10 | 8.41 ( 4.52 - 15.65 ) | 3.07 ( 2.19 ) | bile acids increased* | 62 | 3091.21(2312.33-4132.46) | 11.14(9.47) |
|  | blood cholesterol abnormal | 10 | 10.38 ( 5.58 - 19.33 ) | 3.37 ( 2.5 ) | red blood cell count decreased* | 57 | 34.33(26.41-44.62) | 5.07(3.41) |
|  | lipids increased* | 9 | 22.89 ( 11.87 - 44.14 ) | 4.5 ( 3.58 ) | blood alkaline phosphatase increased* | 56 | 59.1(45.35-77.02) | 5.85(4.19) |
|  | drug screen false positive | 9 | 26.74 ( 13.86 - 51.6 ) | 4.72 ( 3.81 ) | neutrophil count decreased | 52 | 20.57(15.63-27.05) | 4.34(2.67) |
|  | creatinine renal clearance decreased | 8 | 10.7 ( 5.34 - 21.44 ) | 3.41 ( 2.45 ) | lymphocyte count increased* | 51 | 257.45(194.52-340.75) | 7.95(6.28) |
|  | cd4 lymphocytes increased* | 8 | 214.2 ( 103.74 - 442.28 ) | 7.61 ( 6.61 ) | white blood cell count increased* | 46 | 26.08(19.49-34.9) | 4.68(3.02) |
|  | t-lymphocyte count decreased* | 8 | 49.47 ( 24.55 - 99.69 ) | 5.6 ( 4.62 ) | cd8 lymphocytes increased* | 43 | 12450.34(7836.31-19781.12) | 12.33(10.63) |
|  | blood creatine increased | 7 | 7.73 ( 3.68 - 16.24 ) | 2.95 ( 1.92 ) | aspartate aminotransferase increased | 43 | 19.1(14.13-25.81) | 4.24(2.57) |
|  | viral load | 7 | 230.87 ( 106.08 - 502.45 ) | 7.71 ( 6.64 ) | protein total decreased* | 42 | 106.39(78.34-144.48) | 6.7(5.03) |
|  | cd4 lymphocytes abnormal* | 7 | 154.66 ( 71.91 - 332.61 ) | 7.18 ( 6.12 ) | white blood cell count decreased* | 41 | 6.29(4.62-8.55) | 2.64(0.97) |
|  | viral mutation identified | 6 | 5.43 ( 2.44 - 12.11 ) | 2.44 ( 1.35 ) | albumin urine present | 38 | 2102.46(1474.46-2997.93) | 10.72(9.04) |
|  | lipase increased | 6 | 4.52 ( 2.03 - 10.07 ) | 2.17 ( 1.08 ) | platelet count decreased* | 34 | 5.55(3.96-7.77) | 2.46(0.79) |
|  | general physical condition abnormal | 5 | 2.93 ( 1.22 - 7.04 ) | 1.55 ( 0.37 ) | neutrophil count increased* | 34 | 63.93(45.55-89.72) | 5.97(4.31) |
|  | viral load decreased | 4 | 239.5 ( 85.47 - 671.13 ) | 7.76 ( 6.4 ) | haemoglobin increased* | 30 | 110.4(76.9-158.48) | 6.76(5.09) |
|  | waist circumference increased | 4 | 20.78 ( 7.76 - 55.62 ) | 4.36 ( 3.07 ) | t-lymphocyte count decreased* | 28 | 680.05(462.44-1000.05) | 9.29(7.62) |
|  | cd4 lymphocyte percentage decreased* | 4 | 350.03 ( 122.14 - 1003.09 ) | 8.25 ( 6.85 ) | blood urea increased* | 26 | 44.37(30.14-65.32) | 5.45(3.79) |
|  | weight abnormal | 4 | 5.99 ( 2.24 - 15.98 ) | 2.58 ( 1.29 ) | blood cholesterol decreased* | 25 | 213.03(143.08-317.16) | 7.69(6.02) |
|  | blood hiv rna | 4 | 364.03 ( 126.67 - 1046.15 ) | 8.29 ( 6.9 ) | haematuria* | 24 | 13.94(9.33-20.83) | 3.79(2.12) |
|  | drug screen positive* | 3 | 3.3 ( 1.06 - 10.24 ) | 1.72 ( 0.28 ) | haemoglobin decreased* | 22 | 4.22(2.78-6.42) | 2.07(0.4) |
|  | hepatitis b dna increased | 3 | 23.7 ( 7.6 - 73.92 ) | 4.55 ( 3.1 ) | red blood cell count increased* | 21 | 119.69(77.72-184.33) | 6.88(5.21) |
|  | blood testosterone decreased* | 3 | 3.83 ( 1.23 - 11.89 ) | 1.94 ( 0.49 ) | platelet count increased * | 18 | 45.87(28.83-72.98) | 5.51(3.84) |
|  | cortisol abnormal* | 3 | 52.91 ( 16.84 - 166.23 ) | 5.69 ( 4.23 ) | albumin globulin ratio decreased* | 18 | 1432.4(869.84-2358.8) | 10.26(8.58) |
|  | blood creatinine decreased | 3 | 3.95 ( 1.27 - 12.27 ) | 1.98 ( 0.54 ) | glomerular filtration rate increased* | 17 | 282.6(174.17-458.51) | 8.09(6.42) |
|  | cd4/cd8 ratio decreased* | 3 | 50.18 ( 15.98 - 157.57 ) | 5.62 ( 4.15 ) | blood albumin increased* | 16 | 374.07(226.6-617.52) | 8.48(6.81) |
|  | / | / | / | / | protein total increased* | 15 | 350.6(209.07-587.93) | 8.39(6.72) |
|  | / | / | / | / | blood hiv rna increased | 13 | 98.72(57.09-170.69) | 6.6(4.94) |
|  | / | / | / | / | white blood cells urine positive* | 10 | 68.35(36.66-127.45) | 6.08(4.41) |
|  | / | / | / | / | urine bilirubin increased* | 10 | 3557.09(1707.18-7411.57) | 11.31(9.57) |
|  | / | / | / | / | blood glucose decreased* | 8 | 3.92(1.96-7.84) | 1.97(0.3) |
|  | / | / | / | / | glucose urine | 7 | 1110.65(505.87-2438.5) | 9.94(8.24) |
|  | / | / | / | / | high density lipoprotein increased* | 6 | 107.23(47.91-240.01) | 6.73(5.05) |
|  | / | / | / | / | t-lymphocyte count increased* | 5 | 493.35(200.35-1214.84) | 8.87(7.17) |
|  | / | / | / | / | red blood cells urine positive* | 5 | 80.44(33.33-194.14) | 6.31(4.64) |
|  | / | / | / | / | urine ketone body present* | 5 | 53.37(22.14-128.63) | 5.73(4.06) |
|  | / | / | / | / | globulins increased* | 5 | 30.92(12.84-74.44) | 4.94(3.28) |
|  | / | / | / | / | blood creatinine decreased | 4 | 20.56(7.7-54.88) | 4.36(2.69) |
|  | / | / | / | / | blood uric acid decreased* | 4 | 151.76(56.45-407.96) | 7.22(5.54) |
|  | / | / | / | / | red cell distribution increased* | 4 | 27.92(10.46-74.54) | 4.8(3.13) |
|  | / | / | / | / | blood magnesium increased* | 4 | 78.91(29.47-211.29) | 6.29(4.62) |
|  | / | / | / | / | cd8 lymphocytes decreased* | 3 | 350.34(110.47-1111.09) | 8.4(6.7) |
| metabolism and nutrition disorders | diabetes mellitus* | 63 | 4.56 ( 3.56 - 5.84 ) | 2.18 ( 1.82 ) | hyperlipidaemia* | 12 | 32.26(18.29-56.92) | 5(3.33) |
|  | lactic acidosis* | 18 | 2.29 ( 1.44 - 3.63 ) | 1.19 ( 0.53 ) | / | / | / | / |
|  | hyperglycaemia* | 17 | 2.46 ( 1.53 - 3.96 ) | 1.3 ( 0.62 ) | / | / | / | / |
|  | obesity | 11 | 3.32 ( 1.84 - 5.99 ) | 1.73 ( 0.89 ) | / | / | / | / |
|  | increased appetite* | 10 | 2.81 ( 1.51 - 5.22 ) | 1.49 ( 0.62 ) | / | / | / | / |
|  | dyslipidaemia | 8 | 7.83 ( 3.91 - 15.67 ) | 2.96 ( 2 ) | / | / | / | / |
|  | abnormal weight gain | 6 | 3.31 ( 1.49 - 7.38 ) | 1.73 ( 0.63 ) | / | / | / | / |
|  | hyperlipidaemia* | 6 | 4.13 ( 1.85 - 9.19 ) | 2.04 ( 0.95 ) | / | / | / | / |
|  | hypertriglyceridaemia* | 5 | 3.96 ( 1.65 - 9.52 ) | 1.98 ( 0.8 ) | / | / | / | / |
|  | hypercholesterolaemia* | 5 | 2.89 ( 1.2 - 6.96 ) | 1.53 ( 0.35 ) | / | / | / | / |
|  | type 1 diabetes mellitus* | 5 | 3.86 ( 1.61 - 9.29 ) | 1.95 ( 0.77 ) | / | / | / | / |
|  | abnormal loss of weight* | 4 | 3 ( 1.12 - 7.99 ) | 1.58 ( 0.29 ) | / | / | / | / |
|  | glucose tolerance impaired* | 4 | 3.49 ( 1.31 - 9.32 ) | 1.8 ( 0.51 ) | / | / | / | / |
|  | fat redistribution* | 4 | 92.87 ( 34.17 - 252.42 ) | 6.48 ( 5.16 ) | / | / | / | / |
|  | hyperuricaemia* | 4 | 4.82 ( 1.81 - 12.86 ) | 2.27 ( 0.97 ) | / | / | / | / |
| musculoskeletal and connective tissue disorders | myalgia | 67 | 2.02 ( 1.59 - 2.57 ) | 1.01 ( 0.66 ) | osteoporosis* | 37 | 14.18(10.25-19.61) | 3.81(2.14) |
|  | osteoporosis* | 17 | 1.66 ( 1.03 - 2.66 ) | 0.73 ( 0.05 ) | osteopenia* | 5 | 4.94(2.05-11.88) | 2.3(0.64) |
|  | rhabdomyolysis* | 15 | 2.03 ( 1.22 - 3.36 ) | 1.02 ( 0.3 ) | / | / | / | / |
|  | muscle atrophy* | 12 | 4.86 ( 2.76 - 8.57 ) | 2.28 ( 1.48 ) | / | / | / | / |
|  | muscle fatigue* | 3 | 3.23 ( 1.04 - 10.03 ) | 1.69 ( 0.25 ) | / | / | / | / |
| neoplasms benign, malignant and unspecified (incl cysts and polyps) | neoplasm malignant* | 81 | 4.66 ( 3.74 - 5.79 ) | 2.21 ( 1.89 ) | acrochordon* | 4 | 78.05(29.15-208.96) | 6.27(4.6) |
|  | kaposi's sarcoma* | 26 | 33.61 ( 22.81 - 49.51 ) | 5.05 ( 4.49 ) | / | / | / | / |
|  | lymphoma* | 19 | 5.53 ( 3.52 - 8.68 ) | 2.46 ( 1.82 ) | / | / | / | / |
|  | squamous cell carcinoma* | 7 | 3.41 ( 1.63 - 7.17 ) | 1.77 ( 0.75 ) | / | / | / | / |
|  | neoplasm* | 6 | 2.29 ( 1.03 - 5.11 ) | 1.2 ( 0.11 ) | / | / | / | / |
|  | hodgkin's disease* | 5 | 5.85 ( 2.43 - 14.06 ) | 2.54 ( 1.36 ) | / | / | / | / |
|  | brain neoplasm* | 5 | 2.43 ( 1.01 - 5.85 ) | 1.28 ( 0.1 ) | / | / | / | / |
|  | throat cancer* | 5 | 3.58 ( 1.49 - 8.6 ) | 1.84 ( 0.66 ) | / | / | / | / |
|  | immune reconstitution inflammatory syndrome associated kaposi's sarcoma* | 4 | 175.02 ( 63.29 - 483.96 ) | 7.34 ( 6 ) | / | / | / | / |
|  | recurrent cancer* | 4 | 3.61 ( 1.35 - 9.63 ) | 1.85 ( 0.56 ) | / | / | / | / |
|  | rectal cancer* | 3 | 4.48 ( 1.44 - 13.9 ) | 2.16 ( 0.72 ) | / | / | / | / |
|  | anal cancer* | 3 | 11.91 ( 3.83 - 37.05 ) | 3.57 ( 2.12 ) | / | / | / | / |
| nervous system disorders | headache | 177 | 1.36 ( 1.17 - 1.57 ) | 0.44 ( 0.22 ) | / | / | / | / |
|  | cerebrovascular accident* | 86 | 3.22 ( 2.61 - 3.98 ) | 1.68 ( 1.37 ) | / | / | / | / |
|  | neuropathy peripheral* | 34 | 1.52 ( 1.09 - 2.13 ) | 0.6 ( 0.12 ) | / | / | / | / |
|  | cognitive disorder* | 19 | 1.89 ( 1.2 - 2.96 ) | 0.91 ( 0.27 ) | / | / | / | / |
|  | epilepsy* | 15 | 2.21 ( 1.33 - 3.68 ) | 1.15 ( 0.42 ) | / | / | / | / |
|  | dementia* | 12 | 1.79 ( 1.01 - 3.15 ) | 0.84 ( 0.04 ) | / | / | / | / |
|  | cerebral infarction* | 11 | 2.44 ( 1.35 - 4.41 ) | 1.29 ( 0.45 ) | / | / | / | / |
|  | nervous system disorder* | 10 | 2.51 ( 1.35 - 4.67 ) | 1.33 ( 0.46 ) | / | / | / | / |
|  | facial paralysis* | 9 | 3.48 ( 1.81 - 6.7 ) | 1.8 ( 0.88 ) | / | / | / | / |
|  | ischaemic stroke* | 8 | 2.23 ( 1.12 - 4.46 ) | 1.16 ( 0.19 ) | / | / | / | / |
|  | sensory disturbance* | 7 | 2.8 ( 1.33 - 5.87 ) | 1.48 ( 0.46 ) | / | / | / | / |
|  | paralysis* | 7 | 2.53 ( 1.2 - 5.31 ) | 1.34 ( 0.32 ) | / | / | / | / |
|  | guillain-barre syndrome* | 6 | 5.88 ( 2.64 - 13.11 ) | 2.55 ( 1.46 ) | / | / | / | / |
|  | sleep paralysis* | 3 | 9.48 ( 3.05 - 29.46 ) | 3.24 ( 1.79 ) | / | / | / | / |
|  | cluster headache* | 3 | 9.57 ( 3.08 - 29.75 ) | 3.25 ( 1.81 ) | / | / | / | / |
|  | bell's palsy* | 3 | 5.47 ( 1.76 - 16.98 ) | 2.45 ( 1 ) | / | / | / | / |
| pregnancy, puerperium and perinatal conditions | abortion spontaneous | 51 | 6.68 ( 5.07 - 8.79 ) | 2.73 ( 2.33 ) | / | / | / | / |
|  | premature delivery* | 12 | 2.5 ( 1.42 - 4.41 ) | 1.32 ( 0.52 ) | / | / | / | / |
|  | pre-eclampsia* | 8 | 8.63 ( 4.31 - 17.29 ) | 3.1 ( 2.14 ) | / | / | / | / |
|  | ectopic pregnancy* | 7 | 15.44 ( 7.34 - 32.47 ) | 3.94 ( 2.91 ) | / | / | / | / |
|  | foetal death* | 7 | 6.07 ( 2.89 - 12.75 ) | 2.6 ( 1.58 ) | / | / | / | / |
|  | stillbirth* | 7 | 7.51 ( 3.58 - 15.78 ) | 2.9 ( 1.88 ) | / | / | / | / |
|  | foetal growth restriction* | 7 | 4.72 ( 2.25 - 9.9 ) | 2.23 ( 1.21 ) | / | / | / | / |
|  | gestational diabetes* | 6 | 6.34 ( 2.85 - 14.13 ) | 2.66 ( 1.57 ) | / | / | / | / |
|  | placenta praevia* | 3 | 24.12 ( 7.73 - 75.24 ) | 4.58 ( 3.12 ) | / | / | / | / |
| psychiatric disorders | insomnia | 121 | 2.4 ( 2 - 2.87 ) | 1.25 ( 0.99 ) | / | / | / | / |
|  | anxiety | 78 | 1.32 ( 1.06 - 1.65 ) | 0.4 ( 0.07 ) | / | / | / | / |
|  | depression | 76 | 1.87 ( 1.49 - 2.35 ) | 0.9 ( 0.57 ) | / | / | / | / |
|  | mental disorder* | 44 | 4.7 ( 3.49 - 6.31 ) | 2.23 ( 1.79 ) | / | / | / | / |
|  | abnormal dreams | 43 | 12.21 ( 9.04 - 16.48 ) | 3.6 ( 3.16 ) | / | / | / | / |
|  | nightmare* | 38 | 6.37 ( 4.63 - 8.77 ) | 2.67 ( 2.2 ) | / | / | / | / |
|  | sleep disorder* | 37 | 2.25 ( 1.63 - 3.11 ) | 1.17 ( 0.7 ) | / | / | / | / |
|  | suicidal ideation | 35 | 2.14 ( 1.53 - 2.98 ) | 1.09 ( 0.61 ) | / | / | / | / |
|  | stress* | 32 | 1.97 ( 1.39 - 2.79 ) | 0.98 ( 0.47 ) | / | / | / | / |
|  | hallucination* | 25 | 1.54 ( 1.04 - 2.28 ) | 0.62 ( 0.05 ) | / | / | / | / |
|  | mood swings* | 15 | 2.94 ( 1.77 - 4.88 ) | 1.55 ( 0.83 ) | / | / | / | / |
|  | paranoia* | 14 | 5.01 ( 2.97 - 8.47 ) | 2.32 ( 1.58 ) | / | / | / | / |
|  | bipolar disorder* | 12 | 6.61 ( 3.75 - 11.65 ) | 2.72 ( 1.92 ) | / | / | / | / |
|  | major depression | 11 | 7.17 ( 3.96 - 12.95 ) | 2.84 ( 2 ) | / | / | / | / |
|  | mood altered* | 11 | 2.2 ( 1.22 - 3.98 ) | 1.14 ( 0.3 ) | / | / | / | / |
|  | psychotic disorder | 10 | 1.95 ( 1.05 - 3.63 ) | 0.96 ( 0.09 ) | / | / | / | / |
|  | loss of libido* | 9 | 5.18 ( 2.69 - 9.96 ) | 2.37 ( 1.45 ) | / | / | / | / |
|  | hallucination, auditory* | 8 | 2.59 ( 1.3 - 5.19 ) | 1.37 ( 0.41 ) | / | / | / | / |
|  | schizophrenia* | 8 | 2.62 ( 1.31 - 5.23 ) | 1.39 ( 0.42 ) | / | / | / | / |
|  | thinking abnormal* | 7 | 2.15 ( 1.02 - 4.51 ) | 1.1 ( 0.08 ) | / | / | / | / |
|  | substance abuse* | 7 | 3.68 ( 1.75 - 7.72 ) | 1.88 ( 0.86 ) | / | / | / | / |
|  | sleep terror* | 6 | 5.67 ( 2.55 - 12.64 ) | 2.5 ( 1.41 ) | / | / | / | / |
|  | post-traumatic stress disorder* | 5 | 2.94 ( 1.22 - 7.07 ) | 1.55 ( 0.38 ) | / | / | / | / |
|  | psychiatric symptom | 5 | 3.44 ( 1.43 - 8.27 ) | 1.78 ( 0.6 ) | / | / | / | / |
|  | schizoaffective disorder* | 4 | 9.72 ( 3.64 - 25.96 ) | 3.28 ( 1.98 ) | / | / | / | / |
|  | near death experience* | 4 | 3.31 ( 1.24 - 8.83 ) | 1.73 ( 0.43 ) | / | / | / | / |
|  | depressive symptom | 3 | 3.75 ( 1.21 - 11.64 ) | 1.9 ( 0.46 ) | / | / | / | / |
|  | negative thoughts | 3 | 5.32 ( 1.71 - 16.52 ) | 2.41 ( 0.96 ) | / | / | / | / |
|  | alcohol withdrawal syndrome* | 3 | 21.2 ( 6.8 - 66.08 ) | 4.39 ( 2.94 ) | / | / | / | / |
|  | neuropsychiatric syndrome* | 3 | 83.23 ( 26.3 - 263.46 ) | 6.33 ( 4.85 ) | / | / | / | / |
|  | communication disorder* | 3 | 4.06 ( 1.31 - 12.6 ) | 2.02 ( 0.57 ) | / | / | / | / |
|  | alcohol abuse* | 3 | 5.35 ( 1.72 - 16.61 ) | 2.42 ( 0.97 ) | / | / | / | / |
|  | dysphemia* | 3 | 3.72 ( 1.2 - 11.53 ) | 1.89 ( 0.45 ) | / | / | / | / |
| renal and urinary disorders | renal failure | 64 | 2.15 ( 1.68 - 2.75 ) | 1.1 ( 0.74 ) | / | / | / | / |
|  | renal impairment | 37 | 1.84 ( 1.34 - 2.55 ) | 0.88 ( 0.41 ) | / | / | / | / |
|  | nephrolithiasis* | 27 | 2.58 ( 1.77 - 3.77 ) | 1.36 ( 0.82 ) | / | / | / | / |
|  | renal disorder | 25 | 2.43 ( 1.64 - 3.59 ) | 1.28 ( 0.71 ) | / | / | / | / |
|  | chromaturia | 16 | 3.9 ( 2.39 - 6.38 ) | 1.96 ( 1.26 ) | / | / | / | / |
|  | renal pain* | 14 | 6.3 ( 3.73 - 10.65 ) | 2.65 ( 1.9 ) | / | / | / | / |
|  | nephropathy | 9 | 3.34 ( 1.74 - 6.42 ) | 1.74 ( 0.82 ) | / | / | / | / |
|  | nephropathy toxic | 9 | 3.59 ( 1.87 - 6.91 ) | 1.84 ( 0.93 ) | / | / | / | / |
|  | polyuria* | 5 | 2.83 ( 1.18 - 6.81 ) | 1.5 ( 0.32 ) | / | / | / | / |
|  | hydronephrosis* | 4 | 2.7 ( 1.01 - 7.19 ) | 1.43 ( 0.14 ) | / | / | / | / |
|  | fanconi syndrome acquired | 4 | 9.72 ( 3.64 - 25.96 ) | 3.28 ( 1.98 ) | / | / | / | / |
|  | glycosuria | 3 | 12.85 ( 4.13 - 39.99 ) | 3.68 ( 2.23 ) | / | / | / | / |
|  | iga nephropathy | 3 | 7.47 ( 2.4 - 23.2 ) | 2.9 ( 1.45 ) | / | / | / | / |
|  | renal tubular injury | 3 | 7.59 ( 2.44 - 23.59 ) | 2.92 ( 1.47 ) | / | / | / | / |
| reproductive system and breast disorders | erectile dysfunction* | 15 | 3.17 ( 1.91 - 5.26 ) | 1.66 ( 0.94 ) | / | / | / | / |
|  | gynaecomastia* | 14 | 2.41 ( 1.43 - 4.08 ) | 1.27 ( 0.52 ) | / | / | / | / |
|  | penile size reduced* | 3 | 16.85 ( 5.41 - 52.48 ) | 4.06 ( 2.61 ) | / | / | / | / |
| respiratory, thoracic and mediastinal disorders | respiratory failure* | 25 | 1.78 ( 1.2 - 2.64 ) | 0.83 ( 0.26 ) | pulmonary sarcoidosis* | 3 | 52(16.71-161.85) | 5.69(4.02) |
|  | pneumothorax* | 8 | 2.27 ( 1.13 - 4.53 ) | 1.18 ( 0.22 ) | / | / | / | / |
|  | pharyngeal inflammation* | 4 | 14.13 ( 5.29 - 37.77 ) | 3.81 ( 2.52 ) | / | / | / | / |
| skin and subcutaneous tissue disorders | rash | 147 | 1.44 ( 1.23 - 1.7 ) | 0.52 ( 0.28 ) | / | / | / | / |
|  | alopecia* | 69 | 1.34 ( 1.06 - 1.7 ) | 0.42 ( 0.08 ) | / | / | / | / |
|  | hyperhidrosis* | 34 | 1.41 ( 1.01 - 1.97 ) | 0.49 ( 0.01 ) | / | / | / | / |
|  | lipodystrophy acquired* | 18 | 37.33 ( 23.43 - 59.5 ) | 5.2 ( 4.53 ) | / | / | / | / |
|  | night sweats* | 15 | 2.19 ( 1.32 - 3.63 ) | 1.13 ( 0.41 ) | / | / | / | / |
|  | rash maculo-papular* | 11 | 2.22 ( 1.23 - 4 ) | 1.15 ( 0.31 ) | / | / | / | / |
|  | drug eruption* | 8 | 2.13 ( 1.07 - 4.27 ) | 1.09 ( 0.13 ) | / | / | / | / |
|  | toxic skin eruption* | 8 | 3.76 ( 1.88 - 7.53 ) | 1.91 ( 0.95 ) | / | / | / | / |
|  | erythema multiforme | 6 | 3.57 ( 1.6 - 7.95 ) | 1.83 ( 0.74 ) | / | / | / | / |
|  | seborrhoeic dermatitis* | 5 | 13.51 ( 5.61 - 32.55 ) | 3.75 ( 2.57 ) | / | / | / | / |
|  | skin odour abnormal* | 5 | 7.3 ( 3.03 - 17.56 ) | 2.86 ( 1.68 ) | / | / | / | / |
|  | lipohypertrophy* | 3 | 20.56 ( 6.6 - 64.07 ) | 4.35 ( 2.9 ) | / | / | / | / |
|  | lipoatrophy* | 3 | 21.74 ( 6.97 - 67.77 ) | 4.43 ( 2.98 ) | / | / | / | / |
|  | erythema nodosum* | 3 | 3.71 ( 1.2 - 11.53 ) | 1.89 ( 0.45 ) | / | / | / | / |
| vascular disorders | peripheral coldness* | 10 | 3.67 ( 1.97 - 6.82 ) | 1.87 ( 1 ) | / | / | / | / |

*indicates unexpected AEs in the algorithm.


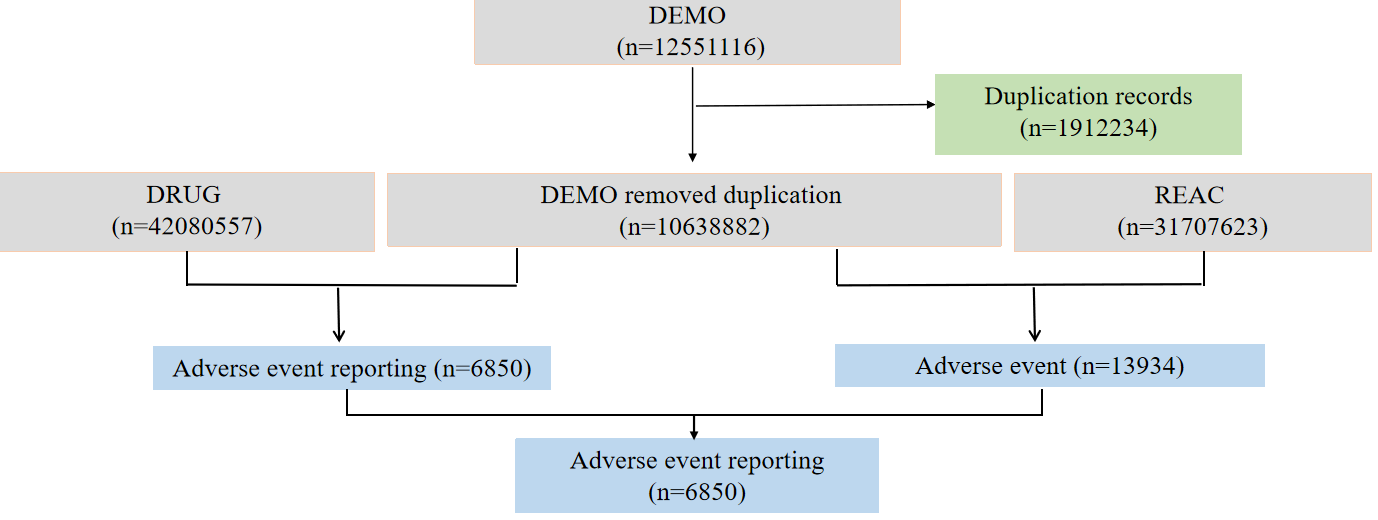


Figure S1 Multistep process of data extraction and processing from the FAERS.
